# Electronic visualized double-lumen endobronchial tube for situs inversus totalis: A case report and literature review

**DOI:** 10.1097/MD.0000000000036295

**Published:** 2023-12-01

**Authors:** Zhi Li, Youyu Wang, Zhiheng Liu, Nanbo Luo

**Affiliations:** a Department of Anesthesiology, Second People’s Hospital of Futian District Shenzhen, Shenzhen, China; b Department of Thoracic Surgery, Inst Translat Med, Shenzhen Second People’s Hospital/The First Affiliated Hospital of Shenzhen University, Shenzhen, China; c Department of Anesthesiology, Inst Translat Med, Shenzhen Second People’s Hospital/ The First Affiliated Hospital of Shenzhen University, Shenzhen, China.

**Keywords:** electronic visual double, lumen endobronchial tube, lung ventilation, single, situs inversus totalis

## Abstract

**Rationale:**

Using an electronic visualized double-lumen endobronchial tube (E-visual DLT) allows for excellent surgical visualization during one-lung ventilation. Situs inversus totalis (SIT) is a rare autosomal recessive genetic condition wherein the bronchial and pulmonary lobar structures on the left and right sides of individuals are reversed compared to those of the general population. In the case of SIT, placing a left-sided E-visual DLT into the right bronchus might offer more advantageous one-lung ventilation. However, there have been no reported instances of using E-visual DLT single-lung ventilation anesthesia techniques for SIT.

**Patients concerns:**

We present a case report detailing the effective implementation of a visualized single-lung ventilation technique under general anesthesia in a 36-year-old male diagnosed with SIT. The patient had a mediastinal mass and underwent thoracoscopic resection of the mediastinal mass using a left-sided approach.

**Diagnoses:**

Based on the findings from the contrast-enhanced chest computed tomography (CT) results, the patient was diagnosed with SIT along with a mediastinal mass. Surgical intervention was proposed to alleviate the cardiac compression caused by the mass. Nevertheless, the administration and handling of anesthesia posed a notable challenge since clinical anesthesiologists encounter contradictory data and a limited number of evidence-based guidelines.

**Interventions:**

Convened a multidisciplinary meeting prior to the initiation of anesthesia to formulate a comprehensive strategy. Throughout the anesthetic management, our team ensured meticulous monitoring, delivered sufficient oxygenation, and established hemodynamic equilibrium. The anesthesia team deliberated and devised a plan to employ a left-sided E-visual DLT placement through the right bronchus for right-sided one-lung ventilation in the patient with SIT. The process of anesthesia induction was subjected to repeated simulations to guarantee patient safety.

**Outcomes:**

Due to the meticulous and effective administration and supervision of anesthesia, the surgery was completed as planned. Subsequently, the removal of the E-visual DLT was executed without any complications.

**Lessons:**

Data and literature about SIT are scarce, necessitating thorough pre-planning and preparation.

## 1. Introduction

Mediastinal mass refers to a nonmalignant neoplasm growing within the mediastinal region.^[1]^ These masses typically do not rapidly spread to other areas, but they may exert pressure on surrounding structures, leading to symptoms or discomfort.^[2]^ The methods for treating benign mediastinal mass may vary based on the mass type, size, symptoms, and overall health of the patient. Typically, surgical resection or thoracoscopic surgery is employed, necessitating one-lung ventilation under general anesthesia during the procedure to ensure an optimal surgical workspace.^[3]^

Situs Inversus Totalis (SIT) is a rare congenital anomaly characterized by the reversal of thoracic and abdominal organs along the sagittal plane, where the heart is located on the right side, and the liver on the left, with the left and right lungs and bilateral main bronchi exchanging positions.^[4]^ At present, regarding the operative strategy for one-lung ventilation in patients with SIT, only one case of single-lung ventilation in an SIT patient has been reported by Bougaki M,^[5]^ focusing on fiberoptic bronchoscope guidance instead of the recently developed and more widespread electronic visualized double-lumen endobronchial tube (E-visual DLT) technique. Anesthesiologists require evidence-based protocols to address the challenges of one-lung ventilation in SIT patients. To our knowledge, we are the first to share our successful experience in managing one-lung ventilation in an SIT patient using an E-visual DLT.

## 2. Case report

A 36-year-old male patient (170 cm, 55 kg) was admitted on July 21, 2023. The patient reported the discovery of a mediastinal mass during a physical examination that had been ongoing for 10 days.

**Figure 1.**
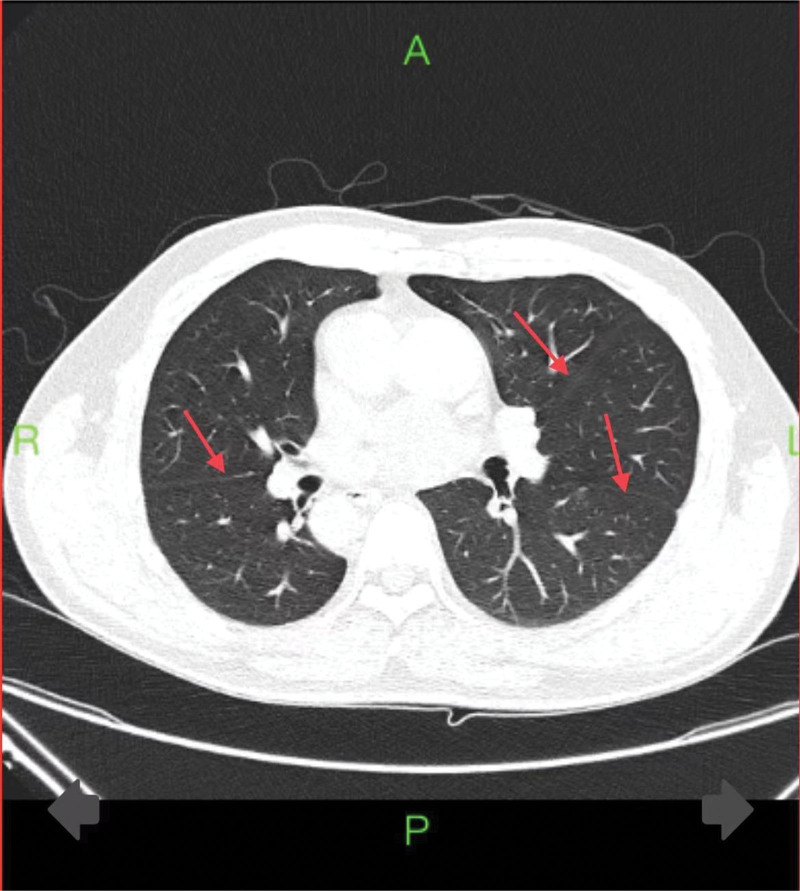
Chest plain scan after contrast-enhancement shows the right lung divided into 2 lobes by the oblique fissure, and the left lung divided into 3 lobes by both the horizontal and oblique fissures, with complete inversion of the left and right main bronchi.

**Figure 2.**
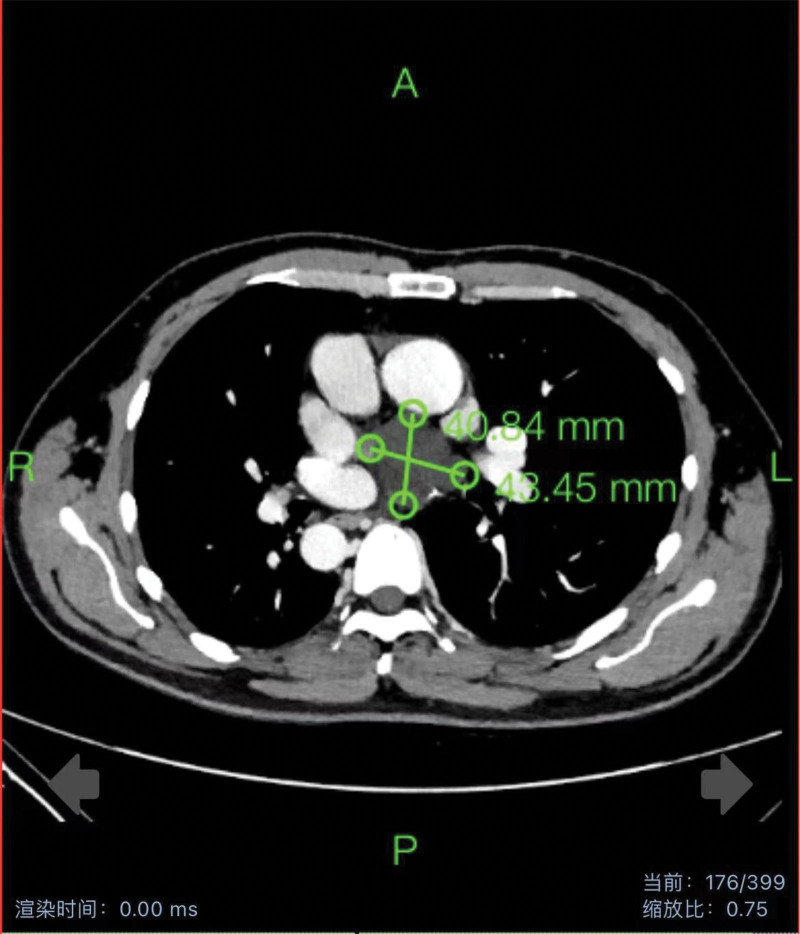
Chest contrast-enhanced CT scan findings: Post-contrast CT scan of the chest reveals a cystic lesion below the bifurcation of the mediastinal trachea, measuring approximately 41*43mm in size.

Enhanced chest CT findings revealed a mirror-image dextrocardia. The left lung was divided into 3 lobes by horizontal and oblique fissures (Fig. 1). Both the left and right main bronchi and the major blood vessels entering and leaving the heart were reversed in position. In the mediastinum, there was a cystic lesion adjacent to important blood vessels and nerves, measuring approximately 43*41mm (Fig. 2), with a suspected benign nature.

The pathological nature of the mediastinal mass was not yet clear, but it exerted a mass effect on nearby structures. The thoracic surgery team decided to perform a thoracoscopic resection of the mediastinal mass to determine the pathology definitively and prevent the mass from compressing surrounding tissues.

However, the anesthesia team considered the complete SIT in the patient, where the lungs and the left and right main bronchi were entirely reversed. The right main bronchus was longer and only contained 2 lobes. Additionally, the left E-visual DLT had a longer safe zone compared to the right-sided E-visual DLT. Consequently, the anesthesia team decided to use the left electronic visualized E-visual DLT for placement into the right main bronchus.

The standard procedures for placing an E-visual DLT for one-lung ventilation in the general population might not necessarily apply to SIT. Implementing one-lung ventilation in SIT patients requires detailed planning and repeated simulations to ensure the success and effectiveness of one-lung ventilation. The planned procedure was the thoracoscopic resection of the mediastinal mass via a left-sided approach under general anesthesia with E-visual DLT placement for one-lung ventilation, scheduled for July 26, 2023.

**Figure 3.**
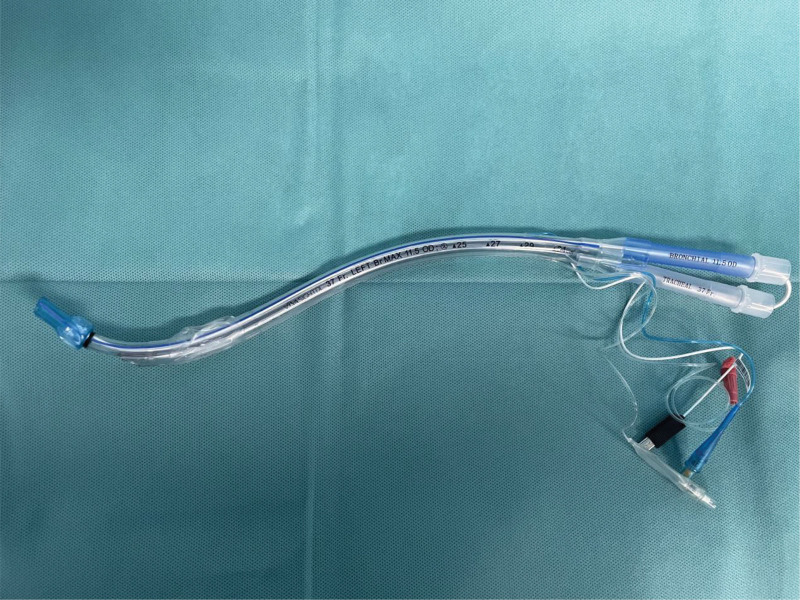
Reshaping of the left E-visual DLT. E-visual DLT = electronic visualized double-lumen endobronchial tube.

**Figure 4.**
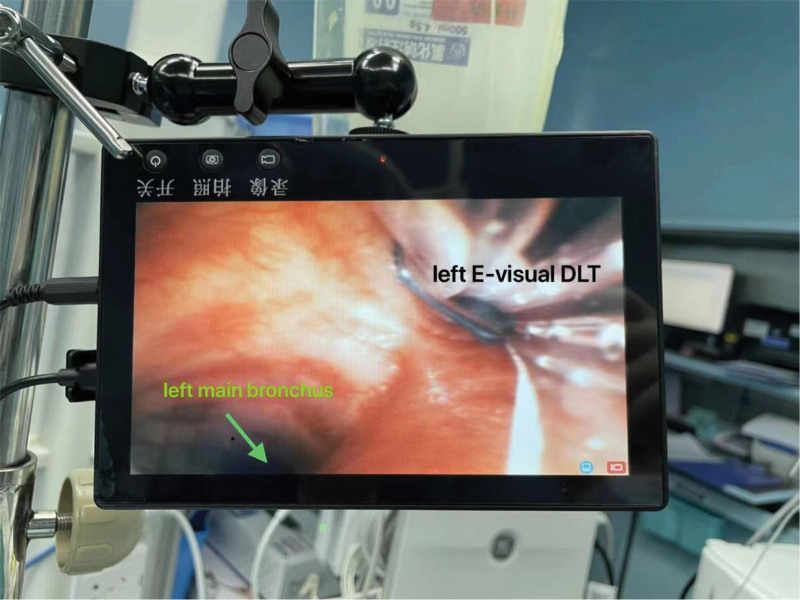
The screen of the left E-visual DLT is flipped, displaying the environment and objects within it in the correct orientation. E-visual DLT = electronic visualized double-lumen endobronchial tube.

Upon arrival in the operating room, noninvasive monitoring was initiated. While breathing room air, the blood pressure measured 130/88 mm Hg, heart rate was 100 beats per minute, and pulse oxygen saturation was 98%. The anesthesia team prepared a disposable sterile Fr37 left-sided E-visual DLT (Guangzhou Weili Medical Instruments Co., Ltd). The E-visual DLT camera and display screen were activated. The left-sided bronchial end of the tube was turned toward the right side, causing the E-visual DLT camera view to invert on the display screen, presenting an inverted image of the operating room environment and equipment. The display screen was then adjusted to show a positive picture of the environment and equipment, and the tube was manually bent in the opposite direction (Fig. 3). Anesthesia induction for general anesthesia was achieved with sufentanil, rocuronium, and propofol. The 37Fr left-sided E-Visual DLT was inserted into the trachea using a laryngoscope. The left E-visual DLT was rotated clockwise while simultaneously monitoring the openings of the patient bilateral main bronchi on the display screen. This facilitated easy positioning of the E-visual DLT bronchial end into the right main bronchus (Fig. 4). After repeated monitoring to confirm effective one-lung ventilation, oxygen (70%, 1.5 L/min) and sevoflurane were maintained. One-lung ventilation proceeded smoothly throughout the surgery with excellent ventilation. Upon completion of the surgery, extubation was uneventful, with no anesthesia-related complications such as airway soft tissue injury, throat pain, or difficulty in speaking. The surgical and anesthesia durations were 150 minutes and 215 minutes, respectively. Intraoperatively, the total blood loss was 20 mL, urine output was 500 mL, and a total of 1500 mL of lactated Ringer solution was administered.

## 3. Discussion

Situs inversus totalis (SIT) is a rare congenital anatomical variation, first documented by Fabricus in 1600 and subsequently named by Sherk^[6]^ in the 17th century. SIT is an autosomal recessive genetic condition characterized by a complete mirror-image transposition of thoracoabdominal viscera. Its incidence ranges from 1/8000 to 1/25,000 in the general population.^[7]^

Due to the complete reversal of bronchial and pulmonary lobe structures in SIT, it is not guaranteed that the standard double-lumen tube operating procedures available in the market can fully cater to successful single-lung ventilation and adequate ventilation in cases of complete lung inversion in SIT patients. For instance, using a left-sided E-visual DLT for placement into the left main bronchus (which anatomically tends to be shorter and encompasses 3 lung lobes) in SIT individuals may potentially obstruct ventilation; the insertion of a right-sided DLT into the right main bronchus (which includes 2 lung lobes) during right-sided single-lung ventilation may cause leakage due to the side holes on the right tube. Consequently, the successful execution of single-lung ventilation techniques in SIT patients is infrequent and poses essential challenges, including the selection of electronic E-visual DLT models, alterations to the tube shape, adjustments to screen orientation, and the direction of main bronchial insertion.

Therefore, the successful single-lung ventilation and anesthetic management plan for SIT patients relies on principles generally applied in thoracic anesthesia. Based on reports by Bougaki.^[5]^ and our prior experiences, several critical considerations warrant close attention.

Firstly, Wójcik et al^[8]^ presented a study involving 21 globally published cases of lung cancer in SIT patients, indicating that despite anatomical inversions, SIT cases conform to regular norms. Hence, advocating bronchial blockers as the primary choice for single-lung ventilation in SIT patients is advisable.^[9]^ However, in cases where mediastinal mass are located at the tracheal bifurcation, the use of bronchial blockers may risk intraoperative displacement affecting single-lung ventilation or causing damage. To ensure effective intraoperative ventilation and minimize potential harm, our anesthesia team opted for E-visual DLT for single-lung ventilation in this patient.

Secondly, Bougaki et al^[5]^ suggested that if DLT is required in SIT, the corresponding DLT should be bent posteriorly, with the left-sided DLT inserted into the right main bronchus and the right-sided DLT inserted into the left main bronchus. Considering the longer safety distance in the right main bronchus, clinical anesthesiologists often prefer inserting the left DLT into the right main bronchus, with prior bending of the left DLT.

Thirdly, Oh et al^[10]^ showcased the benefits of E-visual DLT. The utilization of E-visual DLT lowers procedural intricacies, improves precision during placement, and elevates success rates. It allows for continuous monitoring of cannula positioning, reducing the necessity for fiberoptic bronchoscopy usage and minimizing sterilization-related expenses. Comparative studies between E-visual DLT and standard DLT showed that the former exhibited faster bronchial intubation rates and higher first-attempt success rates. However, selecting left E-visual DLT for insertion into the right main bronchus necessitates prior adjustments such as turning the left E-visual DLT to the right, flipping the screen, adjusting camera position, and altering the display direction on the screen. Subsequently, the left E-visual DLT is inserted into the trachea, guided by the image on the screen, and rotated clockwise into the right main bronchus, with the procedure deemed complete once single-lung ventilation efficacy is confirmed.

Finally, a strong recommendation is made to thoroughly research the anatomical features of both lungs and main bronchi in SIT patients before anesthesia and to simulate the specific operational procedures and their details repeatedly. In this case, through pre-anesthetic planning and multiple simulations, the modified left E-visual DLT could be smoothly guided into the right main bronchus under the guidance of the inverted electronic visual camera screen.

## 4. Conclusions

We are the first to report the successful application of E-visual DLT under general anesthesia to achieve single-lung ventilation in SIT patients. Thoracic surgery in SIT individuals is a rare occurrence, necessitating meticulous planning by an expert anesthesia team. Furthermore, when dealing with such procedures, it is crucial to bear in mind the unique anatomical positioning characteristics of both lungs and main bronchi in SIT patients, which pose both challenges and significance in clinical practice.

## Author contributions

**Data curation:** Zhi Li.

**Methodology:** Nanbo Luo.

**Resources:** Youyu Wang.

**Supervision:** Zhiheng Liu.

**Writing – original draft:** Zhi Li.

**Writing – review & editing:** Nanbo Luo.
